# A Top-Down Approach to Infer and Compare Domain-Domain Interactions across Eight Model Organisms

**DOI:** 10.1371/journal.pone.0005096

**Published:** 2009-03-31

**Authors:** Chittibabu Guda, Brian R. King, Lipika R. Pal, Purnima Guda

**Affiliations:** 1 GenNYsis Center for Excellence in Cancer Genomics and Department of Epidemiology & Biostatistics, State University of New York at Albany, Rensselaer, New York, United States of America; 2 Department of Computer Information Science, Mansfield University, Mansfield, Pennsylvania, United States of America; 3 Center for Advanced Research in Biotechnology, University of Maryland Biotechnology Institute, Rockville, Maryland, United States of America; Centre for Genomic Regulation, Spain

## Abstract

Knowledge of specific domain-domain interactions (DDIs) is essential to understand the functional significance of protein interaction networks. Despite the availability of an enormous amount of data on protein-protein interactions (PPIs), very little is known about specific DDIs occurring in them. Here, we present a top-down approach to accurately infer functionally relevant DDIs from PPI data. We created a comprehensive, non-redundant dataset of 209,165 experimentally-derived PPIs by combining datasets from five major interaction databases. We introduced an integrated scoring system that uses a novel combination of a set of five orthogonal scoring features covering the probabilistic, evolutionary, evidence-based, spatial and functional properties of interacting domains, which can map the interacting propensity of two domains in many dimensions. This method outperforms similar existing methods both in the accuracy of prediction and in the coverage of domain interaction space. We predicted a set of 52,492 high-confidence DDIs to carry out cross-species comparison of DDI conservation in eight model species including human, mouse, *Drosophila*, *C. elegans*, yeast, *Plasmodium*, *E. coli* and *Arabidopsis*. Our results show that only 23% of these DDIs are conserved in at least two species and only 3.8% in at least 4 species, indicating a rather low conservation across species. Pair-wise analysis of DDI conservation revealed a ‘sliding conservation’ pattern between the evolutionarily neighboring species. Our methodology and the high-confidence DDI predictions generated in this study can help to better understand the functional significance of PPIs at the modular level, thus can significantly impact further experimental investigations in systems biology research.

## Introduction

Proteins rarely function alone; a vast majority of proteins must interact with other proteins to perform their intended functions. In recent years, determination of protein-protein interactions (referred to henceforth as PPIs) has been at the forefront of systems biology research resulting in high-throughput generation of such interactions for many proteomes of model organisms [1]. When proteins interact, the binding interface of the interaction is generally localized to specific conserved segments of the interacting proteins that are broadly known as domains. These domains create the interface of an interaction through highly specific recognition events. Thus, knowledge on domain-domain interactions (referred to henceforth as DDIs) is very important for understanding the nature and the significance of PPIs. For instance, DDIs have been used to gain a better understanding of protein networks [2], for predicting the effects of mutations [3] and alternative splicing events that effect interacting domains [4], for developing drugs to inhibit pathological protein interactions [5], [6], and for designing novel protein interactions [7].

Despite the availability of abundant PPI data, little is known about interactions at the domain level because the PPI data is available as ‘binary’ data, *i.e.* interaction between a given pair of proteins is either ‘found’ or ‘not found’. In the case of multi-domain proteins, which constitute about 65–70% of the eukaryotic proteomes [8], [9], binary interaction data is not very informative, because it does not reveal which two domains form the binding interface(s) in an interaction. Moreover, it is tedious to determine DDIs using experimental methods; thus, computational methods are essential for inferring domain-domain interactions from the vast amount of available protein-protein interaction data. Deng *et al.* [10] have attempted to infer DDIs from a small number of two-hybrid interactions in yeast (Y2H), using association rules and maximum likelihood estimations (MLE), resulting in low specificity of prediction. Ng *et al.* [11] employed an integrated method to predict DDIs from disparate data sources that include Y2H data from the DIP database, protein complexes from the Protein Data Bank (PDB) and domain fusion data from Rosetta Stone sequences. Another method, known as domain pair exclusion analysis (DPEA), has been developed based on MLE method using DIP data from 68 different species, and domain definitions from the Pfam database [12]. The same dataset was also used to predict DDIs based on a parsimony approach [13], [14]. Nevertheless, a large number of domains of unknown function (DUFs) were used in these studies. Nye *et al.* [15] have developed a statistical approach to measure the strength of evidence for physical contact between domains in interacting proteins. An integrated scoring method that uses multiple scoring criteria with multiple datasets was also reported recently to predict DDIs [16]. Domain interactions have also been inferred from protein structure data using information based on geometric association of domain interaction interfaces [17], conserved binding mode analysis from the docking patterns of interacting domains [18], or co-evolutionary analysis [19]. Hence, it is clear that computational methods for inferring domain-domain interactions have been constantly evolving to integrate and take advantage of the vast amount of updated annotation data emerging in many dimensions.

Several PPI databases from high-throughput experimental studies are available online, including the Database of Interacting Proteins (DIP, http://dip.doe-mbi.ucla.edu), IntAct (http://www.ebi.ac.uk/intact), BioGrid (http://www.thebiogrid.org), BIND (http://www.bind.ca), MINT (http://mint.bio.uniroma2.it/mint) and HPRD (http://www.hprd.org). Though each database uses a different set of criteria for collection and curation of interaction data and each covers a variety of species, there is a significant overlap among them [20]. The quality of predictions generated by any computational method depends squarely on the scoring algorithm and the datasets used for training the method. Most of the current methods for inferring DDIs from PPIs are based on one or a few scoring features that were trained on limited sets of PPI data. In this study, we use a robust PPI dataset representing 2,725 species, and implement a top-down approach based on a probabilistic model using five independent scoring features. The scoring algorithm implemented in this study is based on a novel combination of orthogonal scoring features that could map the interaction propensity of two domains in many dimensions. The proposed scoring features are derived both from tested as well as novel approaches to maximize the prediction accuracy of functionally-relevant interactions, and to efficiently filter out random or irrelevant interactions. Using this method, we predict and analyze DDIs from eight model species to understand the conservation patterns of DDIs across species. A recent study has compared DDI conservation across five species using a small set (∼3000) of structurally known DDIs [21]. In contrast, here we predict a large-scale dataset of over 65,000 high-confidence DDIs, and use these data to perform cross-species comparison of DDIs from eight organisms. To our knowledge, this study is the first of its kind to explore and compare a vast domain interactome space covering a broad evolutionary spectrum of species.

## Methods

### Interacting and non-interacting protein datasets

We created a comprehensive, non-redundant dataset of experimentally-derived interacting proteins by combining multiple datasets (downloaded in the PSI MI 2.5 format) from five major protein interaction databases that include DIP (Database of Interacting Proteins) (http://dip.doe-mbi.ucla.edu/), IntAct (http://www.ebi.ac.uk/intact), BIND (Biomolecular Interaction Network Database, http://www.bind.ca), HPRD (Human Protein Reference Database, http://www.hprd.org/) and MINT (Molecular Interaction database, http://mint.bio.uniroma2.it/mint). These source databases use a combination of ‘spoke’ and ‘matrix’ models for expanding binary PPI interactions from complexes. These datasets were fairly overlapping both within and across databases, and protein sequences in these databases were originally indexed with different source identifiers from UniProt, DIP, GenBank, etc. To remove redundancy, we first created datasets of unique sequences (based on full-length protein sequence string comparison) within each database and then merged them to create a non-redundant dataset of interacting protein sequences, each indexed with our internal identifier. Note that this internal identifier can be used to map all the original identifiers for a given sequence in their source database(s). Finally, we obtained 70,769 unique protein sequences (denoted as *P*) representing 209,165 unique PPIs (denoted as *P_int_*). Note that proteins in *P* still exhibit some level of redundancy because splice variants with minimal sequence differences are included as unique proteins due to the fact that protein-protein interactions are isoform-specific.

Experimental datasets are not available on non-interacting protein pairs. Hence, we generated a set of putative, non-interacting protein pairs (denoted as 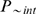) using the proteins in *P*, after excluding the PPIs in 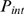 and by imposing certain biologically-relevant restrictions. To assemble this set, we could have assumed that any pair of proteins in *P* that are not present in 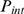 are non-interacting. However, such an approach would generate numerous false negatives given the incomplete nature of 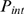, resulting in a very large set of 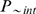. Instead, we imposed two restrictions to define a non-interacting protein pair: first, the two proteins must be present in the same species; second, the two proteins should be localized into different subcellular locations, where the majority of protein-protein interactions are spatially constrained. The subcellular localization information was derived from Swiss-Prot annotations which are based on experimental evidence. Based on these restrictions, we obtained about ∼16.7 million putative negative PPIs in this study.

### Domain Definitions and domain mapping

Domain definitions were used from the InterPro domain database (release 16.0, http://www.ebi.ac.uk/interpro). Some of the InterPro entries are as small as 3 residues that represent active sites, binding sites and prints. We filtered out these entries with a length cutoff of at least 10 residues to eliminate potential noise in the datasets. We mapped the remaining domains onto the set of proteins in *P*.

### Rosetta stone proteins

Rosetta stone proteins are those that have two or more fused domains encoded by a single gene in one species, but whose constituent domains are encoded by separate genes as single domain proteins in the same or another species [22]. In this study, we defined single domain proteins as those with only one InterPro domain annotation and with at most 30 amino acids outside of the domain boundaries on either end of the protein. Based on this criterion, the set of domains occurring in all single domain proteins (denoted as *D_sdp_*) were identified from the entire UniProt protein database (collected by August 7th, 2007). Proteins containing at least two domains from *D_sdp_* were considered as Rosetta stone proteins, and pairs of such domains that co-occur on Rosetta stone proteins were considered as Rosetta domain pairs (RDPs). Out of 4,015,827 protein sequences from UniProt containing InterPro mapping, we found 1,095,580 single domain proteins (27%) corresponding to 9,403 unique InterPro domains. Of these, 7,551 domains were found to co-occur in Rosetta stone proteins in 129,236 binary combinations (RDPs). Out of these, only 34,006 RDPs were found to match with the DDIs used in the current study.

### DDI datasets for testing and comparison

We downloaded a dataset of positive DDIs (based on Pfam database version 21.0) from the iPfam database (http://www.sanger.ac.uk/Software/Pfam/iPfam) [23], which provides known DDIs curated from X-ray crystal structure data in PDB. Predicted DDIs from two recent methods were downloaded to compare against the prediction performance of our method. These include 25,352 DDIs from Lee *et al*'s method [16] that were predicted with a Bayesian likelihood score of 0.25 or more, and 24,625 DDIs from GPE method [14] which were predicted with a probability of 0.25 or more. Both of these methods use domain definitions from the Pfam database. In contrast, our method uses domain definitions from the InterPro database, and a different scoring system that ranges from −12.4 to 38.4. Hence for comparison against the above methods, we used only 147,453 predicted DDIs that have both Pfam domain mapping available and fall in the positive score range (more than zero).

Curated true negative DDIs are required to test the performance of our method, but such experimentally-derived datasets do not exist. Hence, we created a putative set of negative DDIs by considering all the unknown interactions among the domains of iPfam as negative. In other words, the same domain space is used for obtaining both positive and negative DDIs which ensures consistency in feature score coverage for DDIs in both datasets. Initially, we obtained about 3.8 million negative DDIs, of which only 93,129 DDIs had scores available in *D_int_* and hence are usable for testing the current method.

### Notations for datasets

We adopt definitions from set theory, where the term *set* is used when each element in the set is distinct, and the term *collection* is used when the multiplicity of each element is important. For a given set *S*, the notation |*S*| means the cardinality (number of elements) of the set.

- *P_int_* = set of non-redundant PPIs, |*P_int_*| = 209,165.
- *P* = set of all proteins from *P_int_*, |*P*| = 70,769.
- *P_∼int_* = set of non-interacting protein pairs derived from *P*, |*P_∼int_*| = 16,738,143
- *D* = set of domains occurring in *P*, |*D*| = 10,389.
- *D_int_* = set of candidate interacting domain pairs (DDIs) constructed by considering all possible domain pairs that could occur in *P_int_*, |*D_int_*| = 614,579.
- *D_∼int_* = a set of putative non-interacting domain pairs extracted from *P_∼int_* that were also matching with *D_int_*, |*D_∼int_*| = 360,739. Note that *D_∼int_* represents only those DDIs from *D_int_* that exist in *P_∼int_*.
- *f_ij_* – Frequency of domain pair *ij* in *D_int_*.
- *f_∼ij_* – Frequency of domain pair *ij* in *D_∼int_*.
- *f_total_* – Total number of domain pairs in *D_int_*.
- *f_∼total_* – Total number of domain pairs in *D_∼int_*.
- *p_ij_* – Average (expected) probability that domain pair *d_ij_* is an interacting pair, from all protein pairs in *P_int_* where *d_ij_* occurs.
- *p_∼ij_* – Average (expected) probability that domain pair *d_ij_* is a non-interacting pair, from all protein pairs in *P_∼int_* where *d_ij_* occurs.

### Algorithm

**Figure 1:**
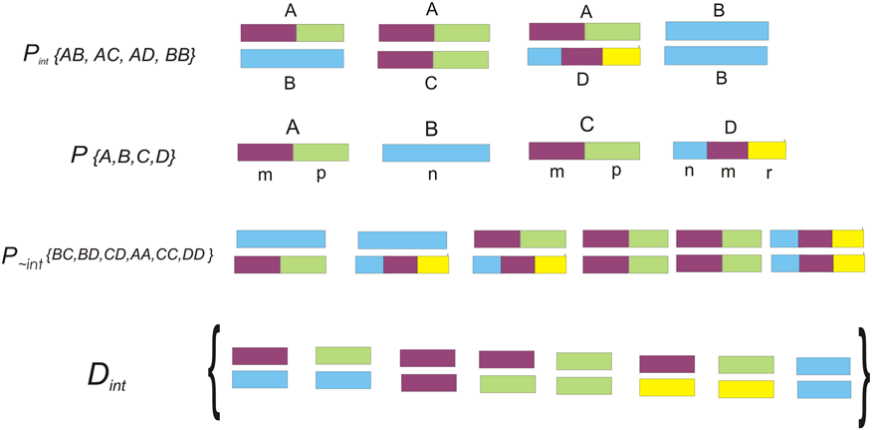
Schematic diagram showing the derivation of datasets. This example shows a set of PPIs, denoted *P_int_*, from which the set of all interacting proteins, denoted *P* are derived. Using *P*, a set of putative non-interacting protein pairs, denoted as *P_∼int_* are generated with some constraints as described in methods. The last section of this figure shows how all possible DDIs (*D_int_*) are derived from *P_int_*. *D_∼int_* is created from *P_∼int_* (not shown in the figure) essentially the same way as *D_int_* is derived from *P_int_*, with an additional step of filtering out domain pairs that do not exist in *D_int_*.

**Figure 2:**
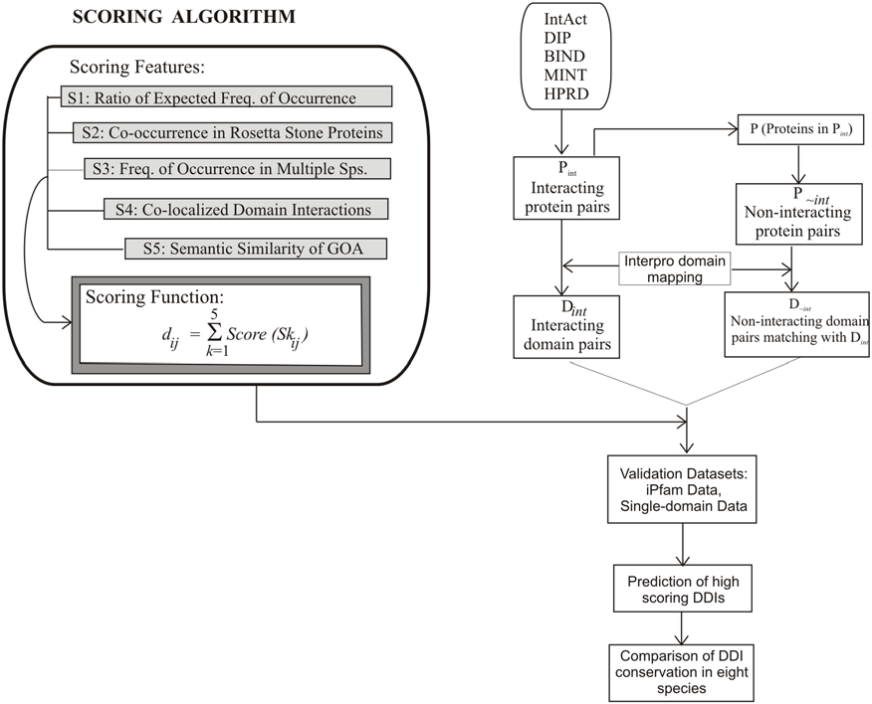
Flow diagram illustrating the scoring system and methodology.

The domain set considered in this study is *D* (|*D*| = 10,389); where in theory, all-to-all domain interactions among *D* can generate ∼54 million unique domain pairs. Nevertheless, such random interactions are not possible in biological systems due to evolutionary, spatial, temporal and functional constraints. The goal of this algorithm is to develop a scoring system that uses a top-down approach for inferring functionally meaningful DDIs from all possible interactions. First, all possible DDIs were extracted from all PPIs in *P_int_*. As illustrated in Figure 1, if *AB*, *AC*, *AD* and *BB* are interacting protein pairs where the domains in respective proteins are denoted as *A(m-p)*, *B(n)*, *C(m-p)* and *D(n-m-r)*, then possible set of DDIs from {*AB*, *AC*, *AD*, *BB*} are {*mn*, *pn*, *mm*, *mp pp*, *mr*, *pr*, *nn*}. It is possible that only *mn*, *pp* and *nn* are true interacting domain pairs in {*AB*, *AC*, *AD*, *BB*}, but this is not obvious from the ‘binary’ interaction data, except for the interaction *BB*. To discriminate functionally relevant DDI(s) in a given PPI from all possible ones, we developed an integrated scoring system that employs five discriminatory features. Each one of these features, denoted *Si*, have some measure of correlation with an aspect of the occurrence of interaction at the domain level. A score is derived for each feature independently. Scores are either based on log ratios between probabilities of features calculated from *P_int_* and *P_∼int_*, or based on probabilities estimated from *P_int_* combined with prior expert knowledge (annotations). We assume that each individual score is an independent descriptor related to the domain pair being scored. Hence, each score reflects the individual probability of one descriptor and the final probability is a product of all five individual probabilities. We use log probabilities to aid in creating a more uniform score, thereby yielding a final score as a sum of the individual log measures.
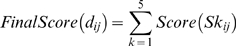
The maximum score for each feature is adjusted at 10 resulting in a maximum final score of 50. See Figure 2 for a pictorial view of the method, illustrating the scoring function and the derivation of datasets. The following section describes the rationale for selecting each scoring feature, and explains how the feature scores (*S1–S5*) for each observed domain pair (*d_ij_*) are derived.

#### *S1* – Ratio of expected frequency of occurrence

Feature *S1_ij_* represents the event that domain pair *d_ij_* is interacting based on the expected frequency of occurrence estimated from a given set of protein pairs. Previous methods that performed similar analysis showed that such a measure can be used to infer DDIs [11], [16], [24]. We derive a score for *d_ij_* based on a ratio of the expected frequency of occurrence in 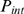 against the expected frequency of occurrence in 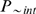.

The expected frequency of occurrence for *d_ij_* is calculated as the average probability of occurrence of *d_ij_* multiplied by the observed number of times that *d_ij_* occurs in the dataset. If a protein contains repeats of the same domain, each unique domain is counted only once for calculation of *d_ij_* probability in a PPI. The probability is estimated empirically from the data. For example, based on the example described in the above paragraph (Figure 1), in PPI *AB*, the two possible DDIs are *mn or pn*. Similarly in PPI *AD*, six DDIs (*mn*, *mm*, *mr*, *pn*, *pm*, *pr*) are possible. So, domain-domain interaction *mn* is possible in multiple PPIs, but with different probabilities. Hence, the probability of occurrence is estimated by calculating the average probability of interaction *d_ij_* over all protein pairs in *P_int_* (or *P_∼int_*) where *d_ij_* occur. We denote this value as *p_ij_* (or *p_∼ij_* when estimated from *P_∼int_*). Before the calculation is complete, all observed frequencies of occurrence *f_ij_* (or *f_∼ij_*) are normalized with respect to the size of each dataset due the disproportionality in the sizes of *P_int_* and *P_∼int_*, resulting in a value between zero and one. The score for *S1_ij_* is then calculated as:
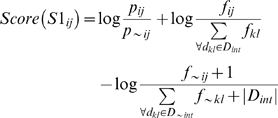
(1)This score gives a log ratio of the expected number of times that putative DDI *d_ij_* will be found in the set of interacting proteins, against the number of times that the same pair is expected to be found in the set of non-interacting proteins. It was developed with the idea of balancing the average probability of a single occurrence of a domain pair against the actual frequency of occurrence over interacting and non-interacting protein datasets. We normalize each *S1_ij_* score to a maximum of 10 by dividing with the maximum *S1_ij_* value observed in the set and by multiplying the result with 10.

A problem with this calculation arises when determining a score for a domain pair *d_ij_* that does not occur in *P_∼int_*. Normally, this would cause the average probability *p_∼ij_* to be zero. In this case, the first term in Equation (1) is replaced by:
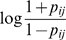
The value of 1−*p_ij_* has the desired property of simulating a probability estimate for *p_∼ij_* that is inversely proportional to *p_ij_*, thus allowing a ratiometric score to be determined even if the domain pair did not occur in *P_∼int_*. In this event only, we limit the maximum observed value for *p_ij_* to be 0.99 in order to prevent the score from dominating the other scores and to prevent a divide by zero error. This problem also causes the observed frequency *f_∼ij_* to be zero. To overcome this problem, we apply the Laplace correction, a well known method used to handle situations where zero frequencies would cause an error in probability estimates [25]. It makes an assumption that all domain pairs in *D_int_* occur at least once in *P_∼int_*, effectively initializing all domain pairs in *D_int_* to have an initial frequency count of one occurrence in *D_∼int_*. This method requires the addition of a corresponding constant in the denominator of the normalization calculation to compensate for this bias and ensure that all normalized values are still between zero and one. This has a desired effect of preventing zero frequency and probability calculation, which would cause errors in the calculation of the log score.

#### S2 – Co-occurrence in Rosetta stone proteins

This feature uses evolutionary reasoning to infer the likelihood of two domains to interact. Fusion of multiple domains on a single polypeptide (known as Rosetta stone proteins) has been considered as the evolutionary solution to facilitate interactions among them. It has been shown that domains that co-occur on Rosetta stone proteins are more likely to interact than those that do not [22], [26].

Let *P_rdp_* be the set of all known Rosetta stone proteins. We say that domain pair *d_ij_* is a Rosetta domain pair if domains of *d_ij_* co-occur in at least one Rosetta stone protein. Let *RDP* be the set of all Rosetta domain pairs determined from *P_rdp_*, and let *RDP_ij_* be defined as the number of proteins in *P_rdp_* where domain pair *d_ij_* is found. We let *S2_ij_* represent the event that domain pair *d_ij_* is interacting based on the occurrence of *d_ij_* in *P_rdp_*. Each *d_ij_* has an observed number of occurrences in *P_rdp_*, with the possible range of 0–52996. A simple function of *RDP_ij_* is used to simulate a probability density function for the probability of *d_ij_* interacting based on information about its occurrence in Rosetta stone proteins. This probability, denoted P(*S2_ij_*), is calculated as follows:
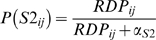
The constant α_S2_ was set to a value of 5.0 based on a simple empirical analysis of observing the behavior of the score over a wide range of values. This value approximately corresponds to the median frequency of occurrence of *d_ij_* in *P_rdp_*. This has the effect of returning probability estimates that are directly proportional to its frequency of occurrence in Rosetta stone proteins. The resulting probability values are multiplied by 10 to allow a maximum score of 10 for *S2*.

#### S3 – Frequency of occurrence in multiple species

This measure is based on the assumption that if domain pair *d_ij_* occurs in interacting proteins from multiple species, then there is an increase in the likelihood that the pair is potentially interacting. This measure also helps to contain the detrimental effects of false positives and false negatives that exist in the PPI datasets, as evidence is being drawn from the interaction data of multiple species. Thus, the effect of a false negative protein interaction in one species can be nullified by accurate data if the same interaction is found in many other species, and similarly for false positive data.

Let *S3_ij_* represent the event that domain pair *d_ij_* is interacting based on the number of occurrences of *d_ij_* in multiple species in *P_int_* and *P_∼int_* , denoted as *#species_ij_* and *#∼species_ij_*, respectively. The score for *S3_ij_* is estimated as a ratio of the occurrence of species in *P_int_* and *P_∼int_*. For consistency, each frequency of occurrence over observed species is converted to a probability distribution. The value of *#∼species_ij_* can be zero for some domain pairs in *D_int_* that do not occur in *P_∼nt_*, which causes an error in the score calculation. To handle this, we apply the Laplace correction in the same manner as in score S1. For each domain pair *d_ij_*, the score for *S3* is based on a log ratio, estimated as:
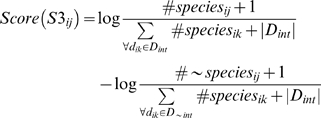
The majority of PPIs in *P_int_* were observed from only 6 model species that include human, mouse, *Drosophila*, *C. elegans*, yeast and *E. coli*, while a small portion of PPIs were observed from a much larger range of species. The resulting log ratio was multiplied by a fixed constant to allow a maximum score of 10 for *S3*.

#### S4 – Co-localized domain interactions in subcellular space

Co-localized domains in a subcellular organelle are likely to interact more often than those localized across different organelles, because they are generally associated with a common pathway or a sub-pathway that is specifically carried out by that organelle. For instance, more than a dozen domains associated with ATP synthesis (ATPases) are found only in mitochondria of eukaryotic animal cells (with the exception of vacuolar ATPase domain, IPR008388) because they are needed only in mitochondria for energy production. The domain datasets used in our previous study [27] show that about 76% of all eukaryotic domains are localized to only one subcellular organelle (see Table S1).

We developed a method for the prediction of protein subcellular localization, called ngLOC [28]. It uses a Naïve Bayes classification model based on frequent *n*-gram occurrence in the primary structure of a protein. The ngLOC method generates a probabilistic measure that a protein sequence is localized into a specific location in a cell. We extended the ngLOC method to predict the localization of proteins over 10 subcellular locations in animal cells, 12 locations in plant cells, five locations in Gram negative and four in Gram positive bacterial cells. These four evolutionary groups are loosely referred to as kingdoms in this work. We let *K* represent the set of kingdoms. For any given kingdom in *K*, we assume a distinct set of subcellular localization classes that are appropriate for that kingdom.

Letting *S_k_* represent the set of all subcellular localization classes for kingdom *k*, we use the ngLOC method to generate a separate probability distribution over *S_k_*, where *P*(*scl_mk_*|*d_i_*) denotes the probability that domain *d_i_* is localized into location *scl_mk_*∈*S_k_* . The problem with prediction of subcellular localization at the domain level is that the same domain can exist on multiple proteins which can be localized to different subcellular locations. We solved this problem by taking an average probability of the predictions from all sequences or subsequences representing a given domain, and renormalizing to ensure the new predictions still represent a probability space, i.e., 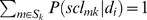. We will refer to this probability distribution as a “subcellular profile” for a given domain.

Our goal is to calculate a single measure that can capture the similarity between subcellular profiles for any arbitrary domain *d_i_* and *d_j_*. First, we sum the absolute value of the difference between the probabilities of each localization. If *d_i_* and *d_j_* have an identical probability distribution, then the difference between them is 0. If they are predicted to localize into different locations with probability of 1, then the measure would be 2. Then we use simple arithmetic (multiply by 0.5) to scale the measure to be between 0 and 1, where 1 implies 100% similarity. We calculate a similarity measure between two domains *d_i_* and *d_j_* in kingdom *k* as:
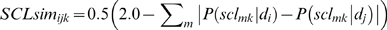
This measure was chosen as it allows similarity to be captured based on the probability over all localization classes considered, and not solely on the most likely prediction. The theoretical possible range for *SCLsim_ijk_* is between 0–1, where a value of 1 implies that domains *d_i_* and *d_j_* have 100% identical subcellular profiles, and a value of 0 implies that domain *d_i_* and *d_j_* are both predicted to localize into entirely different compartments with a probability of 1.

For each prediction for domain *d_i_*, ngLOC outputs two confidences scores: *CS*(*d_i_*) – a confidence measure for the most probable prediction, and *MLCS*(*d_i_*) – a confidence measure for the likelihood that *d_i_* is localized into multiple organelles. The first score is equivalent to the probability of the predicted localization. The second score was developed to aid in predicting when a protein is multi-localized as well as which compartments the sequence is localized into. This is important, as we showed that about 24% of domains localize into more than one organelle (Table S1). We chose a CS threshold of 40 and an MLCS of 60 to obtain only predictions of moderate to high confidence for use in building the subcellular profile for each domain.

Using the subcellular profile, we recalculate the CS and MLCS according to the same calculation employed in the ngLOC method. Using these confidence scores, we calculate a weight for each domain pair *d_ij_* in kingdom *k*, denoted *wSCL_ijk_*. The purpose of the weight is to allow high confidence predictions to have more influence on the final score than moderate confidence predictions. For domain pair *d_ij_* in kingdom *k*, we first determined *minCS_ijk_* – the minimum confidence score observed between *CS*(*d_i_*) and *CS*(*d_j_*) in kingdom *k*. Similarly, we also determined *minMLCS_ijk_* – the minimum MLCS value observed for both predictions. The weight is then simply computed as the maximum of *minCS_ijk_* and *minMLCS_ijk_*:
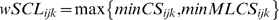



Finally, we can factor in the number of occurrences in each kingdom because, for each domain *d_i_*, we know the frequency of occurrence that the domain was found to occur in each of the four kingdoms separately. We incorporate this information in score S4 by first calculating a probability density modeling the event that any arbitrary domain *d_i_* in *D* occurs in kingdom *k*. This is computed as the fraction of instances of domain *d_i_* that are found in species belonging to kingdom *k*.
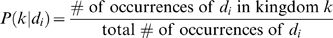
Using this value, we can compute P(*k*|*d_ij_*) as the probability that domain pair *d_ij_* is found in kingdom *k*. We can make a simplifying assumption that the probability of any two arbitrary domains being found in a particular kingdom are independent. Then, P(*k*|*d_ij_*) is simply:
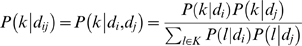
Using these calculations, the final calculation for score S4 is computed as follows:
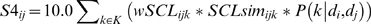
This function was designed to allow high score values that are in proportion to high similarity measures which are captured through comparison of the subcellular profiles between *d_i_* and *d_j_*. The *P*(*k*|*d_i_*, *d_j_*) term can counteract the similarity measure if both domains are found in entirely different kingdoms. The design criteria is that if the subcellular localization for each domain is predicted to be two distinct locations with high confidence, then they are not likely to interact, and thus the integrated score should be reduced accordingly. The multiplier 10.0 is used for scaling purposes, thereby allowing a maximum score of 10.

#### S5-Semantic similarity of GO annotations

This scoring feature was implemented based on the semantic similarity of GO annotation between the two domains in domain pair *d_ij_*. The idea is that if two domains are associated with similar cellular processes and/or involved in similar function, they are more likely to interact. This concept has been successfully used for determining the functional similarity of PPIs [29]. The GO annotation (GOA) for a domain can be based on three concepts i.e., biological process (P), molecular function (F) and cellular component (C). In this scoring feature, we use only the first two concepts (P and F). Concept C is similar to the subcellular location feature used for score S4, and hence is not used here. Since each InterPro domain has a specific function, finding identical GOA for two interacting domains is unlikely, unless it is a homotypic interaction, i.e. interaction between two identical domains. Therefore, we compute a semantic similarity measure between the GO terms of interacting domains within a GO concept, using the method reported by Brown and Jurisica [29].

The GO terms associated with all InterPro domains were obtained from ‘Interpro2Go’ mapping (an expert-curated mapping available at http://www.ebi.ac.uk/interpro). The probability of minimum subsumer, *P_ms_* was determined separately for biological process (P) and molecular function (F) GO concepts using the following derivation. Let *g_1_* and *g_2_* represent the set of GO terms from domains *i* and *j*, respectively; let *S(g_1_, g_2_)* represent the set of shared parental GO terms of *g_1_* and *g_2_*, and let Gc represent GO concept P or F. Then, *P_ms_* is calculated as the minimum frequency of occurrence of the set of shared GO terms over biological process or molecular function concepts:
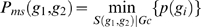
A similarity measure based on this probability is then calculated as the negative log probability of minimum subsumer, using the following equation.
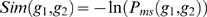



In brief, the similarity score between two GO terms is higher if they share a common parent with a more specific GO term (less frequent), and vice versa. The total similarity score is the sum of similarity scores from concepts P and F. Hence, S5 can range from 0–10, where 0 indicates no match in GO terms and 10 indicate perfect match of GO terms in both concepts.

### Conservation similarity of domains or DDIs across species

We derive a similarity measure between all pair-wise combinations of species based on the predicted proportion of domains or DDIs that are shared between species, by using the basic probability set theory. Let *A* and *B* represent the set of DDIs that were predicted to interact in species A and B, respectively. The union of these two sets represents our sample space of all domain pairs that are predicted to interact in either species A or B, denoted as the set *A* ∪ *B*. We assume a uniform probability distribution across the sample space. The number of domain pairs that are shared between two species is the intersection of A and B, denoted as *A* ∩ *B*. The probability of each species is then the fraction of domain pairs in that particular set over all domain pairs in the union, where the size of the union is determined as: 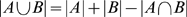.

Using the sample space of predicted interacting domain pairs between species A and B, we assume a uniform probability over the sample space, and assume that 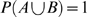. Basic set probability allows us to derive a similarity measure for two species A and B, called *Sim*(*A*,*B*), as:
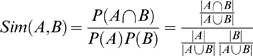
This was adopted from a measure in information theory called “specific mutual information”, which gives the relative amount of information shared between two random variables when these variables take on specific values [30], and is defined as:
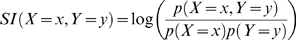
In the context of this study, it is interpreted as the amount of information shared between two specific species, based on the shared DDIs or domains observed between these species. The value of *Sim*(*A*,*B*) will always be between 0 and 1, where 0 implies no similarity between A and B, and 1 implies that the two species have 100% identical domain interaction patterns over all possible interactions observed between the two species.

## Results

### Interaction datasets and domain mapping

We created a comprehensive, non-redundant dataset of experimentally-derived PPIs by combining data from five major interaction databases that include BIND, DIP, HPRD, IntAct and MINT. This dataset contains 70,769 unique interacting protein sequences (denoted as *P*) from 2,725 species, representing 209,165 unique binary PPIs (denoted as *P_int_*). Domain definitions were used from the InterPro domain database, which provides the most comprehensive, expert-curated set of protein domains. Out of 70,769 proteins in *P*, 58,999 (83.4%) have at least one InterPro domain mapped in our study. Thus, out of 209,165 PPIs in *P_int_*, only 166,259 are usable, *i.e.* where each partner protein has at least one InterPro domain mapping with a domain length of at least 10 amino acids. The InterPro database contains 15,064 entries, of which about 69% (10,389 domains) were found at least once in our comprehensive dataset, covering over two-third of the entire domain space.

Experimentally-determined negative PPIs are not available other than a few sporadic literature-based instances. Hence, we generated a set of putative non-interacting protein pairs (*P_∼int_*) from the proteins in *P_int_*, using criteria that minimize the likelihood of interaction between a selected protein pair (see methods). Additionally, *P_∼int_* is established over similar protein space and domain space as *P_int_*, which justifies the ratiometric feature scores obtained from *P_int_* and *P_∼int_*. Sets of all possible DDIs (denoted as *D_int_*) and putative non-interacting domain pairs (denoted as *D_∼int_*) were derived from *P_int_* and *P_∼int_*, respectively. The size of *D_int_* is 614,579 while the size of the original *D_∼int_* is about 8.4 million; however, there are only 360,739 DDIs at the intersection of *D_int_* and the original *D_∼int_*, which are the useful ones for calculating ratiometric feature scores for *P_int_* against *P_∼int_*. Hence, the size of usable *D_∼int_* is only 360,739.

### Scoring algorithm

We selected five orthogonal scoring features that include: (*S1*) ratio of expected frequency of occurrence, (*S2*) co-occurrence in Rosetta stone proteins, (*S3*) frequency of occurrence in multiple species, (*S4*) co-localized domain interactions in subcellular space, and (*S5*) semantic similarity of GO annotations. The maximum value for each feature score is set at 10 to ensure even contribution of each score to the final integrated score. Scores *S1* and *S3* are determined as ratios of feature values in *P_int_* versus *P_∼int_*, while *S2*, *S4* and *S5* are calculated from the feature values of the domain pair in consideration. The sum of these five scores gives the integrated score (IS) for each DDI in a given PPI. Note that only *S1* and *S3* scores could be determined for the entire set of DDIs in *D_int_*, while the coverage of *S2*, *S4* and *S5* varies widely across *D_int_*. The coverage for *S2* is rather low at 5.5%, while *S4* and *S5* have 79.6% and 39.2% coverage, respectively. The reason for the sparse nature of these scores is due to the fact that they are based on the evolutionary (*S2*), spatial (*S4*) and functional (*S5*) knowledge attributed to domains from experimental studies, and such information is not available for all protein domains. As a result, only 1.8% (11,269) of the *D_int_* has a score available for all the five features.

### Validation of prediction performance

We used two positive datasets and one putative negative dataset of DDIs for validating the prediction performance of our method. The first positive dataset contains DDIs from the iPfam database which has been widely used as a ‘gold standard’ for validating predicted DDIs [13], [14], [16], [19], [21]. Out of 4,030 DDIs in the iPfam database, only 3,947 were usable for testing the current method and the rest were eliminated due to lack of InterPro mapping. We have created the second positive dataset from the single-domain PPIs as described in the methods. Since each partner protein in a single-domain PPI has only one domain, corresponding domains are expected to interact (unless the PPI is a false positive). This dataset has 728 overlapping DDIs in the iPfam dataset, which are removed to obtain 2,790 DDIs from the single-domain PPIs. As described in the methods, a set of 93,129 ‘putative negative’ DDIs were used for testing the current method.

First, we compare the performance of individual feature scores and the integrated score using the iPfam DDIs (see Figure S1 for more details on individual feature scores). Due to unbalanced coverage of feature scores across *D_int_*, we used the percentile method to sort the total predictions by each feature score alone, and to compare their accuracy. Prediction accuracy by individual scores was fairly good, ranging from 62–80% in the 80^th^ percentile, while the integrated score has clearly excelled by predicting 86% of the iPfam DDIs in the same percentile, and 77% in the 90^th^ percentile. These results confirm that each one of the selected scoring features (S1–S5) is capable of discriminating functionally relevant DDIs from random interactions.

**Figure 3:**
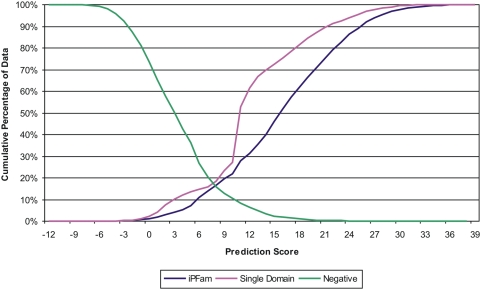
Cumulative distribution of positive and negative test datasets against the entire range of prediction score. Positive test sets include iPfam DDIs and DDIs in single-domain PPIs, while negative test set includes DDIs created from random combination of domains in iPfam excluding the iPfam DDIs.

**Figure 4:**
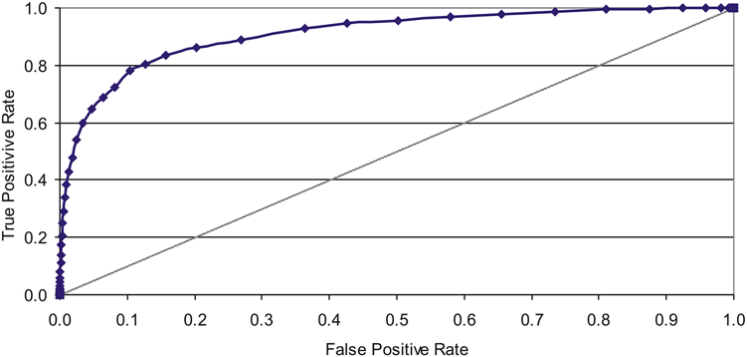
ROC curve plotting true positive rate against false positive rate across the entire range of score thresholds.

Figure 3 shows the cumulative distribution of positive (iPfam and single-domain DDIs) and negative DDIs against the integrated score. At the point where the iPfam and negative DDI lines intersect in this chart (at a score threshold of 8.5), about 85% of the negative DDIs score less than about 85% of the positive iPfam DDIs. The single-domain DDI line shows a similar pattern with slightly lower performance at the upper score thresholds, yet a stronger performance at the lower thresholds compared to the iPfam DDIs. These results clearly demonstrate that the integrated score developed in this study is very effective in discriminating functionally-relevant DDIs from the random pairs. The integrated score will also allow us to predict DDIs at different accuracy levels by adjusting the score threshold. To test the performance of this method at various score thresholds, we created an ROC curve (Receiver and Operating Characteristics) by plotting the true positive rate against the false positive rate (Figure 4). The area under the ROC curve is a good indicator of a good classifier and our ROC curve demonstrates that a large fraction of true positives can be predicted with a low rate of false positives at higher score thresholds.

Table 1 shows the cumulative percentage of score distributions for positive and negative test set DDIs over the entire range of scores. Though the theoretical maximum for the integrated score is 50, we observed that none of the DDIs scored more than 38.4; this is because the score coverage is sparse by some scoring features, or because those DDIs that are covered by all feature scores did not achieve maximum scores from all. As seen in Table 1, most of the DDIs in iPfam and single-domain test sets are predicted at higher score thresholds. For instance, at a score threshold of 9, 80.5% of iPfam and 76.5% of single-domain DDIs were accurately predicted, while only 12.7% of the negative DDIs were falsely predicted at this threshold. On the other end, only 4.3% of the iPfam DDIs scored below 3, while 50% of the negative DDIs scored below the same score threshold. Based on the results shown in Figure 3, Figure 4 and Table 1, we conclude with confidence that there is a significant relationship between the integrated score for a domain pair and the likelihood that the domain pair interacts. For further analysis of DDIs, we used a conservative score threshold of IS > = 10 to select 65,515 top scoring DDIs. This score cutoff roughly corresponds to the top 10 percentile of our predicted DDIs with an accuracy of 78% and a false positive rate of 10%. These predictions along with domain names, scores and species can be accessed from the supporting dataset (Data Sets S1).

**Table 1:** Cumulative distribution of DDIs in iPfam, DDIs from single-domain PPIs and the negative DDIs across the entire score range of all possible DDIs in D_int_.

Score Threshold	<−6	−6	−3	0	3	6	9	12	15	18	21	24	> = 27
Cumulative % of iPfam DDIs	100.0	100.0	99.8	98.8	95.7	89.0	**80.5**	68.6	54.0	38.2	24.9	13.7	6.0
Cumulative % of single-domain PPIs	100.0	100.0	99.8	97.7	89.8	85.1	**76.5**	38.5	27.6	18.6	10.7	5.9	2.4
Cumulative % of negative DDIs	100.0	99.2	92.4	73.5	50.1	26.8	**12.7**	6.4	2.4	1.0	0.4	0.2	0.0

Each column shows the cumulative % of data scoring the value in that column and higher. The entire range of all observed scores fell between −12.43 and 38.37.

### Comparison of prediction accuracy against existing methods

**Figure 5:**
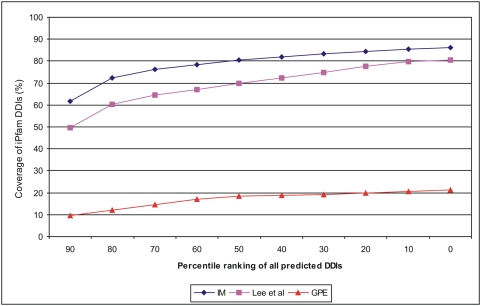
Comparison of the prediction accuracy of the current integrated method (IM) with Lee *et al*'s method and GPE method.

We compared the performance of our method against two recently published methods that include a generalized parsimonious explanation (GPE) method [13], [14] and an integrated method by Lee *et al.* [16]. Prediction results from these three methods can't be compared directly because they use different scoring systems with varying DDI coverage space, and their accuracies were tested against different versions of the iPfam DDI dataset. To make a fair comparison of their predictive performance, we used percentile ranking of all predicted DDIs by each method and adjusted their coverage to the size of the iPfam dataset version used by corresponding method. Figure 5 shows the cumulative fraction of the coverage of iPfam DDIs predicted by each method across different percentile thresholds. Our integrated method (IM) clearly shows superior performance compared to the Lee *et al*'s method, while results obtained from the GPE method are far below. The overall coverage of the iPfam DDIs by our method is 86.2% compared to 80.62% by Lee *et al* and 21.3% by GPE method. Indeed, our method predicted 3,401 iPfam DDIs (86.2% of 3,947) compared to 2,080 (80.6% of 2,580) predicted by Lee *et al*; however, the percentages are calculated with respect to the size of the iPfam version used in each method. These results emphatically suggest that the current integrated method is robust with superior accuracy and coverage compared to the existing methods.

### Prediction of DDIs from eight model organisms

We used a conservative score threshold of IS > = 10 to predict potential DDI(s) in a given PPI of each species. This score roughly corresponds to a true positive rate of 78.1% and a false positive rate of 10.3%. For comparative analysis of DDIs, we selected 8 model organisms including a bacterial and a plant species. These species include human (*Homo sapiens*), mouse (*Mus musculus*), *Drosophila melanogaster*, *Caenorhabditis elegans*, yeast (*Saccharomyces cerevisiae*), *Plasmodium falciparum*, *Escherichia coli*, and *Arabidopsis thaliana*.

Table 2 shows some vital statistics on the analysis of predicted DDIs from eight species. The fraction of usable interacting proteins (those with at least one InterPro domain mapping) varies widely across species from 3.1% to 75.5% due to the unbalanced nature of available experimental PPI datasets. The proteome coverage of usable interacting proteins is excellent in yeast (75.5%) and *E. coli* (74%) followed by *Drosophila* (41.6%), and human (32.4%) presumably due to their extensive use as model organisms. Consequently, these proteins are well annotated resulting in high number of usable PPIs (where each partner protein has at least one InterPro domain mapping) in these species. The domain space covered by interacting proteins is the highest in human (5099 unique domains) followed by *Drosophila*, yeast and *E. coli*. The number of unique DDIs in each species predicted with a score threshold of 10 or more vary widely and there appears to be no correlation between the number of covered domains and the number of predicted unique DDIs. This can be explained by the power law behavior where a few domains have many interacting partners and a majority of other domains interact with only a few partners. The scale-free behavior of domain interaction networks is well documented in the literature [31], [32].

**Table 2:** Species-wise analysis of predicted domain-domain interactions.

Species	1	2		3	4	5	6	
	Proteome size	Usable interacting proteins^a^		Usable PPIs b	Domain coverage in *P_int_* c	Unique predicted DDIs d	Species-specific DDIs^e^	
		Total	%				Total	%^f^
Human	37,993	12,311	32.4	47,837	5,099 (76%)	25,287	14,918	60.0
Mouse	32,745	3,173	9.7	4,258	2,400 (36%)	4,197	304	7.2
*Drosophila*	16,273	6,775	41.6	20,578	3,643 (76%)	7,226	2,568	35.5
*C. elegans*	22,515	2,488	11.1	3,934	2,025 (45%)	2,340	463	19.8
Yeast	5,800	4,381	75.5	43,403	3,303 (90%)	19,083	11,820	61.9
*Plasmodium*	5,250	730	13.9	1,246	828 (36%)	858	218	25.4
*E. coli*	4,330	3,205	74.0	10,589	3,221 (82%)	12,044	9,670	80.3
*Arabidopsis*	35,011	1,070	3.1	2,642	749 (16%)	1,288	383	29.7

a A usable interacting protein is the one that has at least one InterPro domain mapped to it.

b Usable PPIs are those whose partner proteins have at least one InterPro domain mapped on each.

c Percentage value in parenthesis is calculated against the total number of InterPro domains mapped in the entire proteome of each species.

d A score threshold of 10 is used to predict DDI(s) in a given PPI and those DDIs that are unique within a species are selected.

e Species-specific DDIs are those that are found only in a particular species.

f Percentage of species-specific DDIs over the unique predicted DDIs in column 5. This number indicates the extent of preservation of DDIs within a species that were not found in any other species.

We calculated species-specific DDIs that are found exclusively in a particular species, which range from 19.8–80.3%, with the exception of mouse which has only 7.2%. This is presumably because the coverage of PPIs in mouse is very small relative to its proteome size , and mouse is very closely related to human than any other pair of species in this study resulting in a number of DDIs shared between these two species. The converse effect is not true in human because the PPI coverage is over ten-fold higher in human compared to mouse. The highest percentage of species-specific DDIs are preserved in *E. coli* (80.3) followed by yeast (61.9%), human (60%) and *Drosophila* (35.5%). These results appear to be correlated with the PPI coverage in corresponding species.

### Cross-species comparison of domain-domain interactions

The results shown in Table 2 left us with several questions, i.e. are DDIs conserved among species, and if so, to what extent? To understand the patterns of DDI conservation within and across species, we carried out an all-to-all comparison of predicted DDIs across eight species. Table 3 shows a matrix of conserved DDIs between all pairs of species in the upper diagonal. The highest number of DDIs were conserved between human and yeast (5682) followed by human and *Drosophila* (4139) and human and mouse (3759). In general, human, yeast and *Drosophila* showed higher conservation of DDIs with other species, which is at least in part attributed to the higher coverage of PPIs in these species. Yet *E. coli*, despite having the highest coverage of PPIs relative to its genome size, appears to have poor conservation of DDIs with other species. Itzhaki *et al* [21] have reported similar results with *E. coli*, where they compared DDI conservation of *E. coli* against four eukaryotic species. However, it is difficult to draw conclusions based solely on these raw numbers because the experimental coverage space of PPIs is highly unbalanced across these species. Hence, we further computed a similarity measure to accurately determine the similarity of DDI conservation between a pair of species by considering the total DDI coverage space, and the number of species-specific and overlapping DDIs. This measure outputs a value between 0–1, where 1 implies absolute conservation of DDIs between the two species and 0 implies no conservation.

**Table 3:** Cross-species comparison of domain-domain interactions from eight model organisms.

Species	HUMAN	MOUSE	DROME	CAEEL	YEAST	PLAF7	ARATH	ECOLI
**HUMAN**		**3759**	**4139**	**1672**	**5682**	**515**	**669**	**1389**
**MOUSE**	*0.91*		**1603**	**803**	**1230**	**189**	**284**	**215**
**DROME**	*0.64*	*0.52*		**1254**	**2210**	**338**	**387**	**363**
**CAEEL**	*0.73*	*0.47*	*0.62*		**1120**	**217**	**253**	**285**
**YEAST**	*0.46*	*0.34*	*0.39*	*0.51*		**577**	**662**	**1893**
**PLAF7**	*0.61*	*0.26*	*0.42*	*0.32*	*0.68*		**85**	**207**
**ARATH**	*0.53*	*0.27*	*0.34*	*0.28*	*0.53*	*0.16*		**233**
**ECOLI**	*0.16*	*0.07*	*0.08*	*0.14*	*0.24*	*0.25*	*0.20*	

HUMAN-*Homo sapiens*; MOUSE-*Mus musculus*; DROME-*Drosophila melanogaster*; CAEEL-*Caenorhabditis elegans*; YEAST-*Saccharomyces cerevisiae*; PLAF7-*Plasmodium falciparum*; ARATH-*Arabidopsis thaliana*; ECOLI-*Escherichia coli*. The upper diagonal shows the number of overlapping DDIs between specific pairs of species. Values in the lower diagonal represent DDI conservation similarity between specific pairs of species.

The lower diagonal of Table 3 shows the similarity measure between species, which are sorted based on their descending evolutionary order from human to *E. coli*. Hence, the similarity measure values closer to the diagonal correspond to evolutionarily closer species, and values further away from the diagonal correspond to distant species. Despite the unbalanced coverage of PPI datasets for different species, this table shows that the computed similarity values mostly correlate with the expected evolutionary distance between a pair of species. The values closer to the diagonal are higher compared to those away from the diagonal in each column, indicating that the DDI conservation is more prominent between evolutionary neighboring species and the conservation gradually disappears as the species become more distant. Note that the similarity between *E. coli* and any other species is the smallest indicating that the bacterial domain-domain interactions are the least conserved in eukaryotic species. This ‘sliding conservation’ pattern of DDIs along the evolutionary path is expected because domain evolution is thought to be the primary evolutionary force for speciation [33].

**Figure 6:**
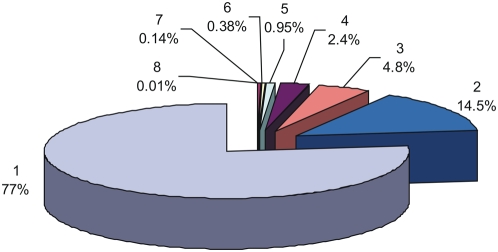
Pie distribution of conserved and non-conserved DDIs in eight model organisms.

We performed a global analysis of 52,492 DDIs that are observed in eight model organisms. Figure 6 shows a pie distribution of conserved and non-conserved DDIs in eight species. About 77% of the DDI analyzed are found in only one species, while 23% are found to be conserved in at least two species and only 3.8% are conserved in at least four species. Of the conserved DDIs, about 63% are found only in two species corroborating the ‘sliding conservation’ pattern explained above. These results suggest that conservation of DDIs across species is rather low despite the fact that corresponding domain conservation similarity is higher (see Table S2). These results led us to examine which DDIs are conserved in more species, and what functions are associated with these conserved DDIs?

### Functional significance of conserved DDIs

We sorted all the conserved DDIs into eight conservation groups based on the number of species containing those DDIs Thus, the first group contains only those DDIs that are conserved in all the 8 species and similarly, the last group contains only those that are found in only one species. Analyzing the functional role of all conserved DDIs is beyond the scope of this study; hence, we looked at the functional role of domains that are associated with the top 5 DDIs in each conservation group. Table 4 lists short descriptions of functions associated with the top five DDIs in each conservation group (ranging from 1–8 species). Most of these conserved DDIs are either homotypic, or those occurring between domains involved in similar functions even if they are heterotypic. As expected, most of these functions are associated with basic cellular processes that maintain genomic integrity (transcription factors, polymerases, etc.) and core metabolism of the cell (kinases, ATPases, etc). For example, the top five DDIs that are conserved in 7 or 8 species include homotypic interactions of Homeodomain-like domain, AAA+ ATPase core domain, Thioredoxin fold domain, etc., and heterotypic interactions among Protein kinase core domain, DNA/RNA helicase C-terminal domain, Map kinase domain, GST- N and C terminal domains. Similarly, domains associated with the top five DDIs conserved in 6 species include several transcription factors, basic helix-loop-helix dimerisation domains, proteosome domains, etc.

**Table 4:** Top five predicted DDIs that are conserved in multiple species, sorted by the number of species conserved.

sps#	Domain-1	Name	Domain-2	Name	Species	Score
8	IPR009057	Homeodomain_like	IPR009057	Homeodomain_like	HMDCYPAE	25.7
8	IPR000719	Prot_kinase_core	IPR001650	DNA/RNA_helicase_C	HMDCYPAE	13.5
8	IPR000719	Prot_kinase_core	IPR003593	AAA+_ATPase_core	HMDCYPAE	13.3
8	IPR003593	AAA+_ATPase_core	IPR003593	AAA+_ATPase_core	HMDCYPAE	11.7
8	IPR012335	Thioredoxin_fold	IPR012335	Thioredoxin_fold	HMDCYPAE	10.5
7	IPR001353	Proteasome_A_B	IPR001353	Proteasome_A_B	HMDCYAE	35.3
7	IPR012287	Homeodomain-rel	IPR012287	Homeodomain-rel	HMDCYAE	31.1
7	IPR004045	GST_N	IPR010987	GST_C_like	HMDCYAE	23.4
7	IPR000719	Prot_kinase_core	IPR003527	MAP_kin	HMDCYPA	22.9
7	IPR004045	GST_N	IPR004045	GST_N	HMDCYAE	20.6
6	IPR004827	TF_bZIP	IPR011700	bZIP_2	HMDCYA	37.4
6	IPR000426	Proteasome_alpha	IPR001353	Proteasome_A_B	HMDCYA	36.9
6	IPR001356	Homeobox	IPR012287	Homeodomain-rel	HMDCYA	36.6
6	IPR001092	HLH_basic	IPR011598	HLH_DNA_bd	HMDCYA	30.5
6	IPR013088	Znf_NHR/GATA	IPR013088	Znf_NHR/GATA	HMDCYE	29.9
5	IPR000243	Pept_T1A_subB	IPR001353	Proteasome_A_B	HMCYA	36.3
5	IPR008331	Ferritin_Dps	IPR008331	Ferritin_Dps	HMDCE	32.5
5	IPR001114	AdlSucc_Synth	IPR001114	AdlSucc_Synth	HMYAE	31.8
5	IPR001163	LSM_snRNP_core	IPR010920	LSM_related_core	HMDCY	31.6
5	IPR009078	Ferritin/RR_like	IPR012347	Ferritin_rel	HMDCE	29.6
4	IPR008946	Nucl_hrmn_rcpt_lig_bd	IPR013088	Znf_NHR/GATA	HMDC	38.4
4	IPR000793	ATPase_a_b_C	IPR004100	ATPase_a_b_N	HYAE	37.3
4	IPR007860	MutS_II	IPR007861	MutS_IV	HCYE	37.1
4	IPR001628	Znf_hrmn_rcpt	IPR008946	Nucl_hrmn_rcpt_lig_bd	HMDC	36.7
4	IPR011261	RNAP_dimerisation	IPR11262	RNAP_insert	HDYE	36.4
3	IPR002398	Pept_C14_p45	IPR011600	Pept_C14_cat	HMD	37.2
3	IPR009025	RNA_pol_RBP11-like	IPR011261	RNAP_dimersation	HDY	36.5
3	IPR013506	Topo_IIA_B_2	IPR013760	Topo_IIA_cen	HYE	36.0
3	IPR009025	RNA_pol_RBP11-like	IPR011262	RNAP_insert	HDY	35.5
3	IPR002205	Topo_IIA_A/C	IPR013757	Topo_IIA_A_a	HYE	35.4
2	IPR002314	AA-tRNA-synt_IIb	IPR015805	His-tRNA_synth	HE	37.4
2	IPR000971	Globin_subset	IPR002337	Haemoglobin_b	HM	34.6
2	IPR001217	STAT	IPR013800	STAT_alpha	HC	34.5
2	IPR009056	Cyt_c_monohaem	IPR012282	Cytochrome_c_R	HY	34.5
2	IPR007120	RNA_pol_Rpb2_6	IPR007644	RNApol_bsu_protrusn	HY	34.3
1	IPR002100	TF_MADSbox	IPR002487	TF_Kbox	A	38.4
1	IPR004516	His-tRNA_synth_IIA	IPR015805	His-tRNA_synth	E	38.3
1	IPR002104	Integrase_cat-core phage	IPR013762	Integrase-like_cat-core_phage	E	37.6
1	IPR007627	RNA_pol_sigma70_r2	IPR014284	RNA_pol_sigma-70	E	36.4
1	IPR001576	Phosphoglycerate_kinase	IPR015824	Phosphoglycerate_kinase-N	Y	35.9

H-human, M-mouse, D-*Drosophila*, C-*C.elegans*, Y-yeast, P-*Plasmodium*, A-*Arabidopsis*, E-*E. coli*.

On the other end, DDIs that are conserved in only one or two species contain domains which are mostly involved in specific functions related to an evolutionary group. For instance, the ‘transcription factor - K box’ domain is found exclusively in plant species and our method predicts that this domain interacts with the ‘transcription factor-MADS box’ domain, only in *Arabidopsis*. Similarly, domains such as Integrase and Integrase-like catalytic core, RNA polymerase sigma-70, etc. are predominantly bacterial domains and interactions between these domains are predicted only in *E. coli*. The Haemoglobin-beta domain is present only in chordates and the interactions between this domain and the Globin-subset domain is predicted only in human and mouse species. The complete list of conserved DDIs with prediction scores, names of partner domains, and the species conserved can be found in the supporting dataset (Data Sets S1).

## Discussion

### Method development

The comprehensive, non-redundant dataset of PPIs compiled by us in this work contains 209,165 PPIs that has an extensive coverage of domain space (two-third of the entire domain/family space in InterPro). To our knowledge, such a large size dataset has not been used before for computational inference of DDIs. This robust dataset has enabled us to develop the current universal method without the biases associated with using smaller datasets that are skewed toward a few species.

Experimentally-determined datasets representing non-interacting protein pairs are virtually lacking; thus limiting the development of negative models by computational methods to compare against the positive datasets. As a result, numerous approaches have been used to establish potential negative PPIs including the use of random protein interactions [11], [21], [34], use of any unobserved interactions at the protein and/or domain level [12], [16], or by assuming a false positive rate and a false negative rate to establish an estimate of negative interaction data in their model [10], [16]. Nevertheless, these approaches treat any unknown interaction as non-interacting, thus potentially label a number of unknown positives as true negatives. In this study, we first generated non-interacting protein pairs (*P_∼int_*) from the same set of proteins (*P*) involved in known interactions (*P_int_* ) after filtering the PPIs in *P_int_* . By doing so, we ascertain that these proteins are expressed (not hypothetical) and involved in at least one known PPI. We then imposed a constraint that the two interacting proteins in a PPI must not be localized to the same subcellular location, where the chances of interaction are higher. This constraint effectively filters out most of the unlikely negative interactions in our putative dataset of negative PPIs.

Despite the origination of both *P_int_* and *P_∼int_* from the same protein set (*P*), the set of PPIs differ between them. Yet, due to the modular architecture of proteins, the same DDI can be found in PPIs belonging to either dataset, but with a different probability. By using the same domain space for comparison, our scores ensure that the probability of interaction between a domain pair in *P_int_* is fairly validated against *P_∼int_*, Hence we are able to effectively contain false positive DDIs from obtaining higher scores as shown in the ROC plot (Figure 4).

The final integrated score developed in this study is a probabilistic measure of five independent scores. Scores are being viewed as random variables, where each variable is modeling the event that some given domain pair will interact. In cases where we can apply negative data from *P_∼int_* to improve our score (i.e. S1 and S3), we can directly compute a log odds ratio, further improving on the ability of the score to distinguish between interacting and non-interacting domains. Despite the fact that the remaining three scores do not use negative data, they are still found to be quite effective at distinguishing between interacting and non-interacting domain pairs (see Figure S1). The range of all scores were scaled between 0–10 to ensure no one score dominated the final score calculation.

Eukaryotic cells have structured subcellular organization; thus, functionally-relevant PPIs, to a larger extent, are expected to occur within their subcellular boundaries. As a consequence, domain interactions are not ‘free-for-all’ in the domain space of a proteome; more often they are under spatial constraints in the cell. This notion is represented by our S4 score (co-localization of domain pairs), which is a novel scoring feature used in this study based on the localization predictions from our ngLOC method [28]. This scoring feature shows one of the best independent performances in predicting iPfam DDIs (Figure S1). Different variants of some of the scoring features used in this study have been used in other studies to infer DDIs [10], [11], [16], [35] or to predict PPIs [29]. Nevertheless, our comprehensive datasets of positive and negative PPIs and our selection of a new combination of scoring features (especially S4) have attributed higher accuracy and coverage to the current method.

We predicted 5,035 homotypic interactions in the 90^th^ percentile that corresponds to 48% of all possible homotypic DDIs in this study (the size of domain set is 10,389). These results corroborate previous observations that homotypic DDIs are very abundant in PPIs [21].

### Comparative analysis of DDIs across model organisms

Comparison of large datasets of DDIs across eight model organisms has revealed interesting insights into the conservation patterns within and across species. About 80% of the high confidence DDIs (52,492 out of 65,515) were also predicted in these eight species giving us abundant DDI space to compare across species. A previous work by Itzhaki *et al.* was the only other similar study, which compared the conservation of about 3000 DDIs across five model organisms [21]. The major methodological difference between these two studies is that we used a top-down approach in contrast to their bottom-up approach. In other words, Itzhaki et al. [21] started with the known DDIs in iPfam [23] and 3DID databases [36], and mapped them to the PPIs across 5 species. In contrast, our method considers all theoretically possible DDIs (|*D_int_*| = 614,579) in the known PPIs, and systematically filtered out random interactions using five scoring features to infer high-confidence DDIs. Table 2 shows that the number of DDIs that are exclusively conserved within a species (species-specific) varies significantly across species. We found that our results have little in agreement with those previously reported by Itzhaki *et al.* We report 80% (*E. coli*), 62% (yeast), 20% (*C. elegans*), 36% (*Drosophila*) and 60% (human) of species-specific DDIs, while Itzhaki *et al.* report 62% (*E. coli*), 18% (yeast), 10% (*C. elegans*), 3% (*Drosophila*) and 41% (human) of such DDIs. The disagreement of results between these two studies may be as a result of a 15-fold difference in the number of DDIs used for analysis in our study.

Due to the incomplete and unbalanced nature of PPI datasets used from different species, it is a challenging task to make a fair comparison of cross-species conservation. The similarity measure we developed in this study to compare the DDI conservation similarity has provided a normalized value to facilitate pair-wise comparisons among all eight species. Cross-species comparison (Table 3) reveals that conservation among species shows a sliding pattern, where neighboring species along the evolutionary path show higher conservation and distant species show lower conservation. The conservation of DDIs across multiple species is very low (Figure 6) given the number of house keeping processes common to all cellular organisms. We speculate that this observation is, at least in part, as a result of the unbalanced coverage of PPI datasets across species, rather than the lack of real conservation. Nevertheless, our results are in accordance with earlier reports; that there is only a small overlap of interactions across multiple species at the PPI level [37], [38] On the other hand, comparison studies using the iPfam database show that a good number of DDIs are conserved across species [21], [39]. Our observation suggesting the lack of reasonable conservation of DDIs across species is based on the use of a large set of high-confidence DDIs (52,492 from 8 species) in contrast to only about 3000 gold-standard iPfam DDIs used by other methods. Additionally, we used a normalized measure to determine the conservation similarity of DDIs to eliminate the bias that originates from unbalanced coverage of PPI datasets across different species. The differences in the data size and in the methodology might explain, at least in part, the discrepancies between our results and those from other studies. The scoring algorithm developed in this study was able to recover 77% of iPfam DDIs in the 90^th^ percentile and about 86% in the 80^th^ percentile; thus helped to identify a large number of high-confidence DDIs that lack structural coverage in the protein data bank.

As expected, DDIs conserved across multiple species appear to perform the essential core of functions to sustain genome integrity, including binding, unwinding, replication, and repair of nucleic acids and other vital enzymatic processes by kinases, proteinases, ATPases, dehydrogenases, and oxido-reductases, etc. (Table 4). We believe that our methodology and the high-confidence DDI predictions generated in this study will help to interpret the functional significance of PPIs, and thus enhance the utility of vast amount of the ‘binary’ protein interaction data generated from high-throughput experiments. Some potential applications of this work include better understanding of PPI networks at the modular level, conferring new functions for domains of unknown function (DUFs), inferring novel protein-protein interactions, etc.

In conclusion, the top-down approach developed in this study is able to predict high-confidence DDIs that will have a multitude of applications in biomedical research. Comparison of the current method with two other methods showed that the prediction accuracy and coverage of our method is strikingly higher. Despite the incomplete and unbalanced nature of available datasets, this method performed very well because (i) the PPI dataset used for training this method is very comprehensive, (ii) the negative PPI dataset contains the same domain space as the positive dataset providing the basis for an effective comparison, and (iii) the scoring algorithm includes a novel combination of orthogonal scoring features that could map the interacting propensity of two domains in many dimensions. Since S2, S4 and S5 scores are based on additional annotation from protein sequences, this method has a high potential to perform better as more experimental annotations become available in the future. This method has generated large-scale datasets of high-confidence DDIs which are effective for cross-species comparison of DDI conservation. This has opened up new opportunities to investigate DDI conservation patterns at various sub-classes of species in the evolutionary spectrum. The methodology and data generated in this project, complemented by experimental validation, can have a significant impact on systems biology research.

## Supporting Information

Table S1 (0.04 MB DOC)

Table S2 (0.04 MB DOC)

Figure S1 (0.14 MB DOC)

Data Sets S1 List of 65,515 high-confidence DDIs predicted by the current method.
(9.95 MB XLS)
