# Supplementary material for: A Top-Down Approach to Infer and Compare Domain-Domain Interactions across Eight Model Organisms
Source: PLoS One. 2009 Mar 31;4(3):e5096. doi: 10.1371/journal.pone.0005096 (PMC2659750; doi:10.1371/journal.pone.0005096)
Supplement: Table S1 — (0.04 MB DOC) [file pone.0005096.s001.doc]

**Table S1.** Distribution of location-specific and shared InterPro domains in nine subcellular locations of eukaryotic non-plant species.

|  | **CYT**  (1052) | **END**  (286) | **EXC**  (774) | **GOL**  (152) | **LYS**  (96) | **MIT**  (674) | **NUC**  (1122) | **PLA**  (809) | **POX**  (101) |
| --- | --- | --- | --- | --- | --- | --- | --- | --- | --- |
| **CYT** | **470** | 94 | 127 | 61 | 20 | 254 | 315 | 92 | 48 |
| **END** |  | **112** | 70 | 35 | 21 | 62 | 76 | 76 | 13 |
| **EXC** |  |  | **495** | 32 | 46 | 40 | 92 | 158 | 13 |
| **GOL** |  |  |  | **40** | 13 | 24 | 48 | 47 | 3 |
| **LYS** |  |  |  |  | **27** | 12 | 11 | 28 | 4 |
| **MIT** |  |  |  |  |  | **343** | 110 | 32 | 52 |
| **NUC** |  |  |  |  |  |  | **737** | 94 | 18 |
| **PLA** |  |  |  |  |  |  |  | **545** | 7 |
| **POX** |  |  |  |  |  |  |  |  | **27** |

CYT-cytoplasm; END-endoplasmic reticulum; EXC-extracellular/secreted; GOL-golgi; LYS-lysosomes; MIT-mitochondria; NUC-nucleus; POX-peroxisomes; PLA-plasma membrane. Total number of domains in each subcellular location is shown in parenthesis in the top row. InterPro domains exclusively found in a subcellular location (location-specific) are shown along the diagonal. The sum of the diagonal is the total number of location-specific domains (2,796) which is 76% of the total number of unique domains in all subcellular locations (3,670). In other words, about 24% of the domains are found in more than one subcellular location. These data are generated from a dataset of experimentally-derived localizations in the Swiss-Prot database used in our previous study [27].
