# Supplementary material for: A Top-Down Approach to Infer and Compare Domain-Domain Interactions across Eight Model Organisms
Source: PLoS One. 2009 Mar 31;4(3):e5096. doi: 10.1371/journal.pone.0005096 (PMC2659750; doi:10.1371/journal.pone.0005096)
Supplement: Table S2 — (0.04 MB DOC) [file pone.0005096.s002.doc]

Table S2: Domain conservation similarity across species.

| Species | HUMAN | MOUSE | DROME | CAEEL | YEAST | PLAF7 | ARATH | ECOLI |
| --- | --- | --- | --- | --- | --- | --- | --- | --- |
| HUMAN |  | 2322 | 3110 | 1796 | 2382 | 720 | 556 | 1107 |
| MOUSE | *0.98* |  | 1783 | 1138 | 1167 | 494 | 431 | 525 |
| DROME | *0.94* | *0.87* |  | 1728 | 2236 | 721 | 526 | 1030 |
| CAEEL | *0.93* | *0.77* | *0.92* |  | 1336 | 560 | 408 | 651 |
| YEAST | *0.85* | *0.67* | *0.88* | *0.80* |  | 720 | 505 | 1198 |
| PLAF7 | *0.89* | *0.68* | *0.90* | *0.77* | *0.90* |  | 234 | 333 |
| ARATH | *0.77* | *0.65* | *0.75* | *0.64* | *0.72* | *0.51* |  | 311 |
| ECOLI | *0.49* | *0.35* | *0.51* | *0.46* | *0.60* | *0.46* | *0.47* |  |

The upper diagonal shows the number of common domains between species. Values in the lower diagonal represents domain conservation similarity between species. HUMAN-*Homo sapiens*; MOUSE-*Mus musculus*; DROME-*Drosophila melanogaster*; CAEEL-*Caenorhabditis elegans*; YEAST-*Saccharomyces cerevisiae*; PLAF7-*Plasmodium falciparum*; ARATH-*Arabidopsis thaliana;* ECOLI-*Escherichia coli*.
