# Supplementary material for: A Top-Down Approach to Infer and Compare Domain-Domain Interactions across Eight Model Organisms
Source: PLoS One. 2009 Mar 31;4(3):e5096. doi: 10.1371/journal.pone.0005096 (PMC2659750; doi:10.1371/journal.pone.0005096)
Supplement: Figure S1 — (0.14 MB DOC) [file pone.0005096.s003.doc]

**Figure S1**. Performance of individual scores (S1-S5) versus the integrated score in predicting iPfam DDIs at different score percentile range. Only those iPfam DDIs that had a score for a given feature were plotted and the fraction of data for each category was determined based on the number of iPfam DDIs containing a score under that category. Percentile values were determined against the entire set of 614,579 domain pairs in *D_int_*.

**Performance of individual scores versus integrated score**

Score S1, the ratio of expected frequency does not require any extraneous knowledge about a DDI other than the average probability of a DDI and the frequency count across all PPIs. To our surprise, the discriminating power of S1 alone is fairly high, with about 63% of iPfam DDIs scoring in the 90^th^ percentile. Score S2 that is based on the Rosetta domain pairs showed a moderate discriminatory power in the 90^th^ percentile, but it correctly predicted about 75% of the Rosetta domain iPfam DDIs in the 80^th^ percentile. Score S3 representing the evidence from multiple species showed the lowest prediction rate at only 53% in the 90^th^ percentile. Score S4 (co-localization of domains in subcellular space) is the second best performer with 65% of iPfam DDIs scoring in the 90^th^ percentile and 80% in the 80^th^ percentile. Finally S5, representing the semantic similarity of GO terms showed the best discriminatory power of all with 68% of the iPfam DDIs scoring in the 90^th^ percentile of all predictions. Finally, the integrated score predicted 77% of the iPfam DDIs in the 90^th^ percentile and 86% in the 80^th^ percentile demonstrating its superiority over individual scores.
